# Community-based self-collected human papillomavirus screening in rural Zimbabwe

**DOI:** 10.1186/s12889-019-6810-5

**Published:** 2019-05-29

**Authors:** Megan B. Fitzpatrick, Ziad El-Khatib, David Katzenstein, Benjamin A. Pinsky, Zvavahera Mike Chirenje, Kathy McCarty

**Affiliations:** 1 Department of Pathology, Stanford University School of Medicine, 300 Pasteur Drive, Stanford, CA 94305 USA; 2 Department of Public Health Sciences, Karolinska Instiutet, Stockholm, Sweden; 3 World Health Programme, Université du Québec en Abitibi-Témiscamingue (UQAT), Québec, Canada; 4 Department of Medicine, Division of Infectious Diseases and Geographic Medicine, Stanford University School of Medicine, 300 Pasteur Drive, Stanford, CA 94305 USA; 5 Biomedical Research and Training Institute of Zimbabwe, 10 Seagrave Rd, Mount Pleasant, Harare, Zimbabwe; 6 Department of Obstetrics and Gynecology, University of Zimbabwe, 630 Churchill Avenue, Harare, Zimbabwe; 7 Chidamoyo Christian Hospital, P.O. Box 330, Karoi, Zimbabwe

**Keywords:** Human papillomavirus, Cervical cancer screening, Cancer screening

## Abstract

**Background:**

In low- and middle-income countries (LMIC), women have limited access to and uptake of cervical cancer screening. Delayed diagnosis leads to poorer outcomes and early mortality, and continues to impede cancer control disproportionately in LMIC. Integrating self-collected, community-based screening for High Risk-Human Papilloma Virus (HR-HPV) into existent HIV programs is a potential screening method to identify women at high risk for developing high-risk cervical lesions.

**Methods:**

We implemented community-based cross-sectional study on self-collection HR-HPV screening in conjunction with existing community outreach models for the distribution of antiretroviral therapy (ART) and the World Health Organization Expanded Program on Immunization (EPI) outreach in villages in rural Zimbabwe from January 2017 through May 2017.

**Results:**

Overall, there was an 82% response rate: 70% of respondents participated in self-collection and 12% were ineligible for the study (inclusion criteria: age 30–65, not pregnant, with an intact uterus). Women recruited in the first 2–3 months of the study had more opportunities to participate and therefore significantly higher participation: 81% participation (additional 11% ineligible), while those with fewer opportunities also had lower participation: 63% (additional 13% ineligible) (*p* < 0.001). Some village outreach centers (*N* = 5/12) had greater than 89% participation.

**Conclusions:**

Integration of HR-HPV screening into existing community outreach models for HIV and immunizations could facilitate population-based screening to scale cancer control and prevention programs in sub-Saharan Africa. Community/village health workers (CHW/VHW) and village outreach programs offer a potential option for cervical cancer screening programs to move towards improving access of sexual and reproductive health resources for women at highest risk.

**Electronic supplementary material:**

The online version of this article (10.1186/s12889-019-6810-5) contains supplementary material, which is available to authorized users.

## Background

Cervical cancer is the third most common cancer globally and disproportionately affects low- and middle- income countries (LMIC) where 80% of new cases occur [1–3]. Cervical cancer can be prevented through screening for and treating precancerous lesions. However, limited uptake of screening and delays in diagnosis lead to early mortality. Opportunistic screening may miss women at the highest risk of cervical cancer, especially women co-infected with HIV in hyper-endemic rural sub-Saharan Africa. In LMIC, the Papanicolaou test (known by “Pap test”) is often not available, so Visual Inspection with Acetic Acid (VIAC) (with/without cervicography) is employed as an alternative cost-effective screening strategy [4]. Regardless of method, the lack of well-trained staff and operator-dependent performance remain limitations to adequate screening [5].

The political and economic environment has influenced the distribution and structure of health provision in Zimbabwe. The HIV/AIDS epidemic had a detrimental effect on health resources in Zimbabwe, especially in rural areas. Since then, great progress has been made towards the World Health Organization’s 90/90/90 goals for HIV testing, treatment, and viral suppression. In fact, the 2015 Zimbabwe Population-Based HIV Impact Assessment found that survey nationwide 86.8% of women knew their status, 87.3% of women living with HIV self-report current use of ART, and 87.9% are virally suppressed [6]. This headway made in HIV care in rural areas is at least in part attributable to successful community-based care combined with hospital care. Multiple studies have found that prevention efforts that combine community health workers (CHW) demonstrate increased uptake of HIV services and treatment adherence [7–9]. CHWs can deliver basic health services and have the added value of established rapport with community members and lower relative human resource cost [10]. CHWs may also be critical in cervical cancer control and prevention in settings where limited transport, health centers, and human resources are barriers to care.

HIV infection is common in rural Zimbabwe and more accessible antiretroviral therapy (ART) means that as more women are living with HIV as a chronic infection. Since High-Risk Human papillomavirus (HR-HPV) is an opportunistic co-infection in the setting of HIV and the cause of > 99% of cervical cancers [11], women living with HIV will also continue to have an increased risk of cervical cancer. The Zimbabwe Population-Based HIV Impact Assessment conducted in 2015–2016 found that HIV prevalence peaks at nearly 30% among women 40–44 years old, coinciding with highest risk of cervical cancer. The high burden of co-infection of HPV among HIV positive women highlights the importance of integrating services for HIV and cervical cancer [6].

The World Health Organization (WHO) has recommended integrated screening for HR-HPV into cervical cancer prevention models worldwide [12]. Cervicovaginal self-collection for HR-HPV is generally well accepted by women [13]. Additionally, a recent meta-analysis found that performance of self-collection is similar to clinician-collected sampling, albeit with a slightly lower sensitivity for detection of HR-HPV [14, 15]. The small decrease in sensitivity is more than compensated by the dramatically increased acceptability and participation rate of self-testing and access among under-screened (served) populations [16, 17]. While most studies have focused on hospital-based or urban self-collection screening, a sentinel cluster-randomized trial in Argentina found a four-fold increase in screening uptake with community-based HPV screening via CHWs in community-based settings [18]. This combination of CHW recruitment and self-collected cervicovaginal samples for HR-HPV screening provides a model to reach and screen women in rural, resource-limited settings. These services can be efficiently combined with existing outreach programs in a scalable comprehensive community care model to increase participation of the most vulnerable populations.

In our study, we describe participation in a community-based self-collection HPV screening program which was combined with existing community outreach models for the distribution of antiretroviral therapy (ART) and immunizations in rural Zimbabwe (World Health Organization Expanded Program on Immunization (EPI)).

## Methods

### Study population

**Fig. 1:**
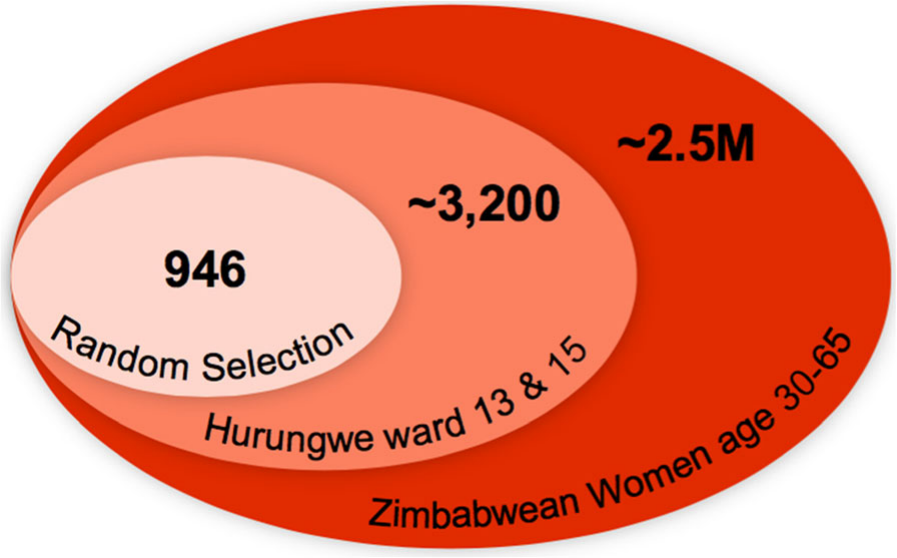
Study population as a percentage of district and country population

The study was conducted in rural Northwestern Zimbabwe in the Hurungwe district in Mashonaland West Province, with the study area defined to be Ward 13/15 which is the approximate catchment area of Chidamoyo Christian Hospital. The estimated population served by Chidamoyo is 32,000 people, with an approximate 3200 eligible women (Fig. 1). From late January 2017 - mid May 2017, we conducted a community-based cross-sectional study. Complete lists of eligible women (age 30–65 years, not pregnant, with an intact uterus) were submitted from community health workers representing a total of 130 villages, and women were selected via random number generation using Microsoft Excel for participation in village-based self-collected HR-HPV testing.

Women were recruited to participate in community outreach days, which were conducted in parallel with scheduled EPI immunization campaigns and ART outreach at 12 rural village center locations. Women recruited in the first 2–3 months of the study were offered a greater number of opportunities to attend, while fewer opportunities were provided to those recruited in March and April recruitment cycles. All women were offered a bar of soap (<$1USD value) as an incentive even if they were disqualified (due to age or pregnancy). Pregnant women and women under 30 years of age were excluded because they are known to have a higher prevalence of HR-HPV infections with complex clearance cycles not observed in non-pregnant women [19, 20]. Women under 30 years old have higher prevalence rates of HR-HPV infection, but lower cervical intraepithelial neoplasia (CIN) and cervical cancer detection rates [21]. HIV status was not used as inclusion or exclusion criterion.

Although formal employment has been challenging in Zimbabwe, rural areas have been somewhat less affected because of sustenance farming and yearly crop growth and sale of cash crops seasonally (tobacco, cotton, maize). The challenges in buying power has resulted in a barter system in rural areas (including hospital fees). The particularly good growing season during the study period could have decreased participation due to increased farm work required.

HR-HPV collection was performed in the community during scheduled outreach visits for provision of ART medications and childhood vaccines. Trained data collectors administered instructions for self-collection in spoken, written and picture/infographic forms and information sessions in Shona, the predominant language and ethnicity in this region of Zimbabwe. The samples were then transported along with medical supplies and personnel to Chidamoyo Christian Hospital for testing using near-care testing using the WHO Prequalified In Vitro Device Cepheid GeneXpert for HPV. Self-collected, cervicovaginal cytobrush specimens were obtained using Cervex brushes collected in ThinPrep PreservCyt (Hologic, Marlborough, MA). Xpert® HPV testing was conducted on a clinic-based GeneXpert (Cepheid, Sunnyvale, CA) in accordance with manufacturer’s instructions within two days to a week of collection, depending on availability of testing platform and electricity. Specimens with invalid results were rerun. If the sample failed twice, the participant was contacted for specimen recollection at a village outreach or Chidamoyo Christian Hospital.

If women were found to have HR-HPV, the clinical coordinator at Chidamoyo notified community health workers and women were invited to attend VIAC on designated days. Of note, there was no VIAC program at Chidamoyo prior to the study, but three nurses and one doctor completed the Ministry of Health VIAC course at the conception of the study to provide these services to all community members.

### Statistical analysis

Statistical analysis was performed using STATA, v. 14 (College Station, TX). A sample size of 700 women was calculated as sufficient to determine a difference for HR-HPV infection/type with a power of 0.80, effect size of .25 and an alpha of 0.05 with proportionate sampling of villages of differing sizes to provide a population representative sample. Basic participation analysis was performed using data entered into an Excel data spreadsheet and STATA, v. 14, and categories of either “no show”, “participated”, “ineligible” or “refused”. Demographic data was only for women who participated in the study via ODK on tablets, and results were downloaded as Excel spreadsheets and basic demographic data was analyzed in STATA, v. 14.

### Study setting

Zimbabwe is bordered by Botswana, Zambia, Mozambique, and South Africa in Southern Africa. An estimated 14 million people live in Zimbabwe, mostly in rural areas [22]. The HIV burden in Zimbabwe is estimated at approximately 14.6% [6], and slightly higher in the adult population and disproportionately affecting women in a ratio of 3:2 [23]. Literacy was at one time the highest in sub-Saharan Africa – in 2015 an estimated 86.5% of the population had basic literacy [22]. Employment rates after the hyperinflation crisis of 2007 remain low, with estimates from 50 to 90% unemployment, however accurate estimates given the current economic conditions are unknown [22].

### Chidamoyo Christian Hospital

The study was conducted in rural Northwestern Zimbabwe in the Hurungwe district in Mashonaland West Province. The study area was defined as Ward 13/15 under the catchment area of Chidamoyo Christian Hospital, with an estimated total population of 32,000 people.

The Chidamoyo Christian Hospital has a 100-bed mission hospital with one operating theater, a labor and delivery ward, pediatric ward, and outpatient clinic which is run by one nurse practitioner (author KM), 1–3 Zimbabwean medical doctors, and approximately 20 nurses, most of whom are “nursing assistants” trained on site. The hospital coordinates immunization outreach under the EPI that was initiated to vaccinate children worldwide by WHO in 1974. The community-based medicine model took an entirely new form in the era of HIV in the Chidamoyo outreach program in Hurungwe. In addition to administration of childhood vaccines in the EPI outreach, Chidamoyo also dispenses antiretroviral medications (ART) to known HIV infected patients on a set weekly schedule of community visits each site every two months.

### Training community health workers

Community health workers were provided with training on cervical cancer prevention and HPV DNA testing, study methods and objectives over three 1-day workshops. Pictorial guides, PowerPoint training, and practice small group interactions were included to help CHWs understand cervical cancer, HPV testing, and how the study was designed. The community health workers provided lists of all eligible women from their villages two months prior to study initiation. Three community members were trained in data collection and instructions for self-collection methods. Female community health workers were offered HPV self-testing to gain personal experience and as a community service, although they were not included in study data. Community health workers informed village chiefs, school heads and other leaders of the community and Ministry of Health (MOH) prior to initiating the study. Community health workers were informed of the women randomly selected in their village, and delivered the news during home visits to explain aims of the study and encourage participation in the scheduled community collection days in their respective villages.

### HIV testing

HIV serologic testing was performed by Ministry of Health certified HIV counselors with the Ministry-provided 3rd generation Alere Determine HIV-1/2 test (Alere/Abbott, Lake Bluff, Illinois, U.S.), a qualitative immunoassay for the detection of antibodies to HIV-1 and HIV2. Reactive specimens were confirmed with the First Response, Rapid HIV 1–2 card test. (Premier Medical Corporation Ltd., Kachigam, India), which is a rapid immuno-chromatographic qualitative test for detection of antibodies to HIV-1 and HIV-2 in whole blood, serum or plasma. The results were interpreted per manufacturer’s instruction document by trained HIV counselors.

## Results

Overall the response rate in the study was 82% (*N* = 778/946), with 12% ineligible due to age under 30, over 65 or pregnancy (*N* = 117/946). Among the 4% (*N* = 40/946) who refused to participate, the most common reason was religious beliefs (42%, *N* = 17/40). Other reasons included relocation, fear of results and/or pelvic exam, and spousal pressure or prohibition. When taken together, only 8% of the women included in the initial recruitment (earliest recruited group) either did not show or refused testing (5% refusal, 3% no show). Very few samples were technically invalid due to inadequate collection or technical difficulty (6/654, 0.9% invalid). Women recruited earliest in the study with more opportunities to participate (average of 3 community visits) had significantly higher participation: 81 and 11% ineligible, while those with fewer opportunities also had lower participation: 63 and 13% ineligible (*p* < 000.1). Participation was also lower during parts of the rainy season (November to April) and early in the harvest season (May to June).

**Fig. 2:**
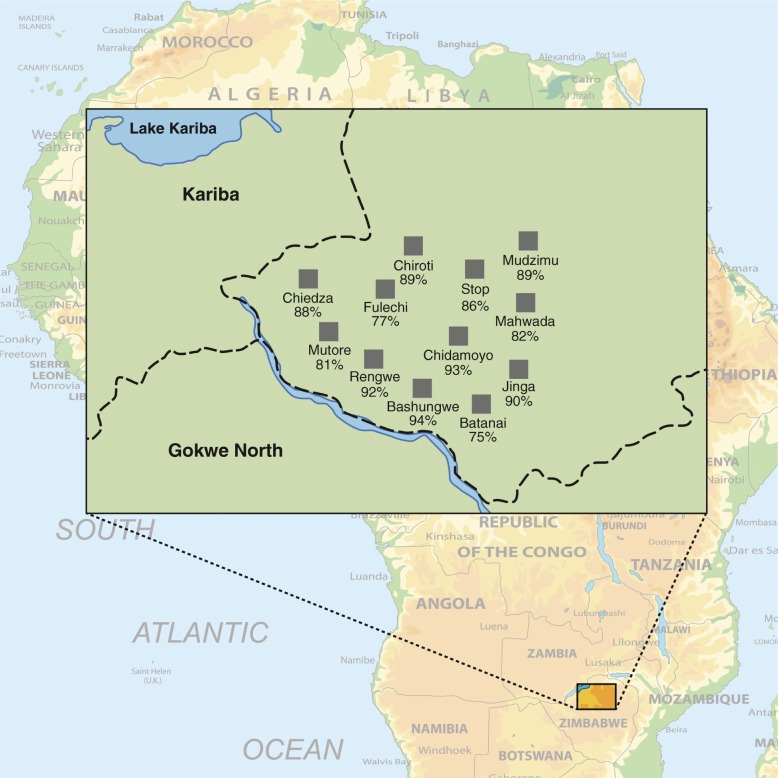
Map of village outreach posts in ward 13 and 15 in Hurungwe district of Zimbabwe with participation rates in the study. Chidamoyo is the hospital center

Some village outreach centers (*N* = 5/12) had greater than 89% participation of the women on the lists, with numerous women not included in the study requesting participation on each day. The villages within walking distance to Chidamoyo Hospital with very active community health workers had participation rates ranging from 89 to 93%. In other areas, further from the hospital and with fewer active community health workers, participation rates were only 75–81% (Fig. 2).

**Fig. 3:**
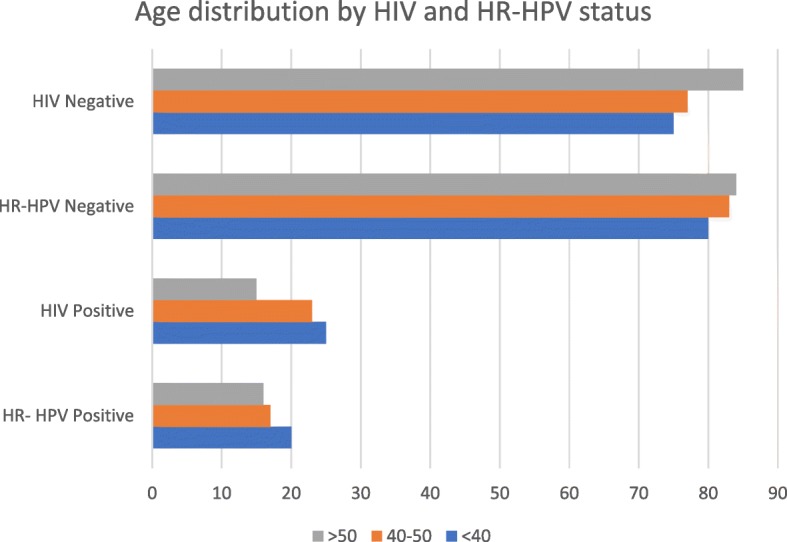
This graph shows the age distribution by age category

The mean age of study participants was 43.6 years old, and average age at first pregnancy was 19.0 and gravidity of 4.47. Half (49.5%) of the women had a grade 7 education, while 24% had no formal schooling. None attended tertiary school. Most women (95.1%) had never been screened for cervical cancer. HIV prevalence was 21.8% in the total population tested (0.3% unknown). HIV prevalence was lowest among women over 50 years old (15%), while women under 40 had the highest rates of HR-HPV detection (20%) (Fig. 3). Many HIV-positive women were already taking ART at the time of the study (90%).

## Discussion

Sub-Saharan Africa has the highest burden of HIV; Eastern and Southern Africa account for 43% of the global total of new infections and an estimated 19.4 million people living with HIV in the region, including 21.8% of women in our study [23]. Data from the 2015 Zimbabwe Demographic and Health Survey found that 14% of Zimbabwean adults (15–49) are HIV positive, with higher prevalence among women (17%) than men (11%) [24]. HIV positive women have a higher risk of carriage of HR-HPV [25] and development of cervical cancer [26]. While cervical cancer screening is critical in HIV infected individuals, it is not currently integrated into HIV care.

Community-based screen and treat programs for HIV have increased initiation of antiretroviral therapy and viral suppression in resource-limited settings [27]. Chidamoyo began combining ART outreach services with childhood immunization campaigns in 2006. Since then, other sexual and reproductive services like family planning have been successfully integrated into these existing outreach models. Integrating HR-HPV testing within this framework could increase access and uptake of cervical cancer screening services. In our descriptive study, 95% of rural women had never been screened for cervical cancer using clinic based approaches (Table 1), while more than 82% of women participated in community-based screening.

**Table 1:** Characteristics of study participants (*N* = 683)

Age (mean years ± SD)	43.6 (10.2)
Age at first pregnancy (mean years ± SD)	19.0 (3.2)
Gravidity (mean ± SD)	4.47 (2.4)
Education	
Did not attend school	163 (24.0%)
O′ Level (Grade 4)	177 (26.1%)
A’ Level (Grade 6)	3 (0.4%)
Grade 7	336 (49.5%)
Tertiary	0 (0.0%)
History of hormonal contraception	480 (70.8%)
Prior screening for cervical cancer	
No (Previously unscreened)	646 (95.1%)
Yes (Previously screened)	33 (4.9%)
Age Categories	
30–39	302 (44.5%)
40–49	186 (27.4%)
50–65	191 (28.1%)
HIV status	
Positive	146 (21.8%)
Negative	521 (77.9%)
Unknown	2 (0.3%)

Effective vaccinations against Human Papillomavirus are available, and will soon be available in Zimbabwe through a partnership with the Global Alliance on Vaccines Initiatives (GAVI) initiated in May of 2018 [28]. Ultimately, a combination of screening for (pre)cancer and primary prevention through vaccination is crucial since these available vaccines are intended to prevent acquisition of the HPV infection in young people, leaving a sizeable vulnerable adult population. In addition, HPV subtypes other than HR-HPV 16/18 are known to circulate and cause cancer in Africa, and may continue to pose risk despite vaccination [3, 5, 25, 29, 30]. Furthermore, adult women currently living with HIV are at higher risk of cervical cancer, requiring continued surveillance [25, 26, 30, 31].

Our program demonstrates a potential method for reaching women for screening. HR-HPV point-of-care testing could be combined with existing community-based programs to improve cancer prevention in Sub-Saharan Africa for women at highest risk [16].

### Study limitations

The participation rates in village outreach self-collected screening were overall promising given the rates of screening at baseline (5% vs. 60–80%). Our study did not have a control population to compare participation rates and was conducted in a region without cervical cancer screening prior to the study. Additionally, given the lack of a comparator group, we were unable to completely exclude confounders that may have increased or decreased participation in screening.

## Conclusions

Our findings suggest that integrating community-based self-collected HR-HPV screening into existing HIV treatment and childhood immunization programs could be considered as a potential method for expanding cervical cancer screening programs into previously challenging to reach rural areas.

A French translation of this article has been included as [see Additional file 1].

A Portuguese translation of the abstract has been included as [see Additional file 2].

## Additional files

Additional file 1: Translation of this article into French. (PDF 247 kb)

Additional file 2: Translation of the abstract of this article into Portuguese. (PDF 99 kb)

## Abbreviations

ART: Antiretroviral therapy
CHW: Community Health Worker *used interchangeably with Village Health Worker
EPI: Expanded Program on Immunization
HIV: Human Immunodeficiency Virus
HR-HPV: High-Risk Human Papilloma Virus
LMIC: Low and middle income countries
USD: United States Dollar
VHW: Village Health Worker
VIAC: Visual Inspection with Acetic Acid and Cervicography
WHO: World Health Organization
