# Supplementary material for: Community-based self-collected human papillomavirus screening in rural Zimbabwe
Source: BMC Public Health. 2019 May 29;19(Suppl 1):603. doi: 10.1186/s12889-019-6810-5 (PMC6538544; doi:10.1186/s12889-019-6810-5)
Supplement: Supplementary file 1 — Translation of this article into French. (PDF 247 kb) [file 12889_2019_6810_MOESM1_ESM.pdf]

BMC Public Health 2019, 19 (Suppl 1) [translated from the original publication]

Dépistage communautaire du papillomavirus humain par auto-prélèvement en zones rurales au Zimbabwe

Megan B. Fitzpatrick[1*], Ziad El-Khatib [2,3], David Katzenstein[4,5], Benjamin A. Pinsky[1,4], Zvavahera Mike Chirenje[6], Kathy McCarty[7]

1. Stanford University School of Medicine, Department of Pathology, 300 Pasteur Drive, Stanford, CA, 94305, USA; megan6@stanford.edu, davidkk@stanford.edu, bpinsky@stanford.edu
2. Department of Public Health Sciences, Karolinska Instiutet, Stockholm, Sweden
3. World Health Programme, Université du Québec en Abitibi-Témiscamingue (UQAT), Québec, Canada, ziad.khatib@gmail.com
4. Stanford University School of Medicine, Department of Medicine, Division of Infectious Diseases and Geographic Medicine, 300 Pasteur Drive, Stanford, CA 94305, USA
5. Biomedical Research and Training Institute of Zimbabwe, 10 Seagrave Rd, Mount Pleasant, Harare Zimbabwe
6. University of Zimbabwe, Department of Obstetrics and Gynecology, 630 Churchill Avenue, Harare, Zimbabwe; mchirenje@uzchs-ctu.org
7. Chidamoyo Christian Hospital, P.O. Box 330, Karoi, Zimbabwe; sistermakate@gmail.com

*Auteur correspondant : Dr. Megan Fitzpatrick
E-mail : megan6@stanford.edu

Résumé

Contexte : Dans les pays à revenus faibles et intermédiaires (PRFI), les femmes ont un accès et un recours limités au dépistage du cancer du col de l'utérus. Le retard de diagnostic entraîne une baisse des chances et une mortalité prématurée et continue à entraver la lutte contre le cancer de manière disproportionnée dans les PRFI. L'intégration d'un dépistage communautaire des papillomavirus à haut risque (HPV-Hr) par auto-prélèvement dans les programmes anti-VIH existants est une méthode de dépistage potentielle permettant d'identifier les femmes à risque élevé de développer des lésions à haut risque du col de l'utérus.

Méthodes : Nous avons mis en œuvre une étude transversale communautaire sur le dépistage des HPV-Hr par auto-prélèvement, en conjonction avec des modèles existants de sensibilisation communautaire pour la distribution de traitements antirétroviraux (TAR) et avec le Programme élargi de vaccination (PEV) de l'Organisation mondiale de la Santé, dans des villages des zones rurales du Zimbabwe entre janvier 2017 et mai 2017.

Résultats : Le taux de réponse globale a été de 82 % : 70 % des répondantes ont participé à l'auto-prélèvement et 12 % étaient inéligibles à l'étude (critères d'inclusion : âge 30-65 ans, pas enceintes, avec un utérus intact). Les femmes recrutées durant les 2-3 premiers mois de l'étude

ont eu plus d'occasions de participer et la participation a donc été significativement plus élevée : participation de 81 % (plus 11 % d'inéligibles), tandis que celles avec moins d'occasions ont également moins participé : 63 % (plus 13 % d'inéligibles) (p<0,001). Certains centres communautaires (N=5/12) ont présenté une participation supérieure à 89 %.

Conclusions : L'intégration du dépistage des HPV-Hr dans les modèles de sensibilisation communautaire existants pour le VIH et les immunisations pourrait faciliter le dépistage et amplifier les programmes de lutte et de prévention contre le cancer en Afrique subsaharienne. Les agents de santé communautaires/ruraux (ASC/ASR) et les programmes de sensibilisation dans les villages offrent la possibilité d'un meilleur accès aux ressources de santé sexuelle et reproductive pour les femmes à haut risque au sein des programmes de dépistage du cancer du col de l'utérus.

Mots clés
papillomavirus humain, dépistage du cancer du col de l'utérus, dépistage du cancer

Contexte

Le cancer du col de l'utérus est le troisième cancer le plus fréquent au niveau mondial et affecte de manière disproportionnée les pays à revenus faibles et intermédiaires (PRFI) qui comptabilisent 80 % des nouveaux cas[1–3]. Le cancer du col de l'utérus peut être évité par le dépistage et le traitement des lésions précancéreuses. Toutefois, le recours limité au dépistage et les retards de diagnostic conduisent à une mortalité précoce. Un dépistage opportuniste peut manquer les femmes présentant le risque le plus élevé de cancer du col de l'utérus, en particulier les femmes co-infectées par le VIH dans les zones rurales hyperendémiques de l'Afrique subsaharienne. Dans les PRFI, le test de Papanicolaou (appelé “test Pap”) est souvent indisponible, on utilise donc l'inspection visuelle avec l'acide acétique (IVA) (avec/sans cervicographie) comme stratégie alternative de dépistage bon marché[4]. Quelque soit la méthode, le manque de personnel bien formé et les performances dépendant de l'opérateur restent des limitations à un dépistage adéquat[5].

L'environnement politique et économique a influencé la répartition et la structure des services de santé au Zimbabwe. L'épidémie de VIH/SIDA a eu un impact délétère sur les ressources de santé au Zimbabwe, en particulier dans les zones rurales. Depuis lors, de gros progrès ont été réalisés dans l'atteinte des objectifs 90/90/90 de l'Organisation mondiale de la

Santé pour le dépistage du VIH, les traitements et la suppression de la charge virale. En fait, le Zimbabwe Population-Based HIV Impact Assessment de 2015 (étude d'impact du VIH réalisée sur la population au Zimbabwe) a montré que 86,8 % des femmes connaissaient leur statut, 87,3 % des femmes porteuses du VIH déclaraient utiliser un TAR et 87,9 % avaient une charge virale nulle[6]. Cette avancée dans les soins liés au VIH, réalisée dans les zones de soins rurales est, au moins en partie, attribuable à des soins communautaires combinés à des soins hospitaliers. Plusieurs études ont montré que les efforts de prévention qui incluent les agents de santé communautaires (ASC) amènent un recours accru aux services liés au VIH et une meilleure observance des traitements[7–9]. Les ASC peuvent délivrer des services de santé basiques et ils ont la valeur ajoutée d'un rapport établi avec des membres de la communauté et d'un coût en ressources humaines comparativement moindre[10]. Les ASC peuvent également jouer un rôle critique dans la lutte contre le cancer du col de l'utérus et sa prévention dans les lieux avec des transports, des centres de santé et des ressources limités qui font obstacle aux soins.

L'infection au VIH est répandue dans les zones rurales du Zimbabwe et un meilleur accès aux traitements antirétroviraux (TAR) signifie que de plus en plus de femmes vivent le VIH comme une infection chronique. Étant donné que le papillomavirus humain à haut risque (HPV-Hr) est une co-infection opportuniste en présence du VIH et la cause de >99 % des cancers du col de l'utérus [11], les femmes porteuses du VIH continueront également à présenter un risque accru de cancer du col. Le Zimbabwe Population-Based HIV Impact Assessment conduit en 2015-2016 a montré que la prévalence du VIH monte à un pic de près de 30 % parmi les femmes âgées de 40 à 44 ans, ce qui coïncide avec le risque maximum de cancer du col de l'utérus. Le taux élevé de co-infections au HPV parmi les femmes séropositives au VIH souligne l'importance d'intégrer des services pour la lutte contre le VIH et le cancer du col de l'utérus[6].

L'Organisation mondiale de la Santé (OMS) a recommandé le dépistage intégré des HPV-Hr dans les modèles de prévention du cancer du col de l'utérus à l'échelon mondial[12]. L'auto-prélèvement vaginal pour le dépistage des HPV-Hr est généralement bien accepté par les femmes[13]. De plus, une méta-analyse récente a montré que les performances de l'auto-prélèvement étaient similaires à l'échantillonnage effectué par un clinicien, malgré une

sensibilité légèrement inférieure dans la détection des HPV-Hr[14, 15]. La faible baisse de la sensibilité est plus que compensée par les taux d'acceptabilité et de participation beaucoup plus élevés avec l'auto-prélèvement et l'accès obtenu parmi les populations sous-dépistées (servies)[16, 17]. Alors que la plupart des études se sont concentrées sur le dépistage en hôpital ou l'auto-prélèvement en zone urbaine, un essai sentinelle randomisé en grappes, réalisé en Argentine, a montré un quadruplement du dépistage des HPV réalisés en communauté via les ASC[18]. Cette combinaison du recrutement d'ASC et de l'auto-prélèvement d'échantillons vaginaux pour le dépistage des HPV-Hr offre un modèle pour atteindre et dépister les femmes dans les zones rurales aux ressources limitées. Ces services peuvent être efficacement combinés à des programmes de vulgarisation existants dans un modèle extensif de soins communautaires complets, afin d'augmenter la participation des populations les plus vulnérables.

Dans notre étude, nous décrivons la participation à un programme de dépistage communautaire des HPV par auto-prélèvement qui a été combiné à des modèles de sensibilisation communautaire existants pour la distribution de traitements antirétroviraux (TAR) et d'immunisations dans les zones rurales du Zimbabwe (Programme élargi de vaccination de l'Organisation mondiale de la Santé (PEV)).

Méthodes

Population de l'étude

L'étude a été conduite dans les zones rurales du nord-ouest du Zimbabwe, dans le district de Hurungwe de la province du Mashonaland occidental, avec une zone d'étude définie comme étant les municipalités (Ward) 13/15 et qui correspond approximativement à la zone desservie par le Chidamoyo Christian Hospital. La population servie par l'hôpital Chidamoyo est estimée à 32 000 personnes, dont environ 3 200 femmes éligibles (figure 1). De fin janvier 2017 à mi-mai 2017, nous avons conduit une étude transversale de nature communautaire. Des listes complètes de femmes éligibles (âgées de 30 à 65 ans, pas enceintes, avec un utérus intact) ont été soumises par des agents de santé communautaires pour un total de 130 villages, et les

femmes ont été sélectionnées par attribution aléatoire d'un numéro avec Microsoft Excel, pour participer dans leur village à un dépistage des HPV-Hr par auto-prélèvement.

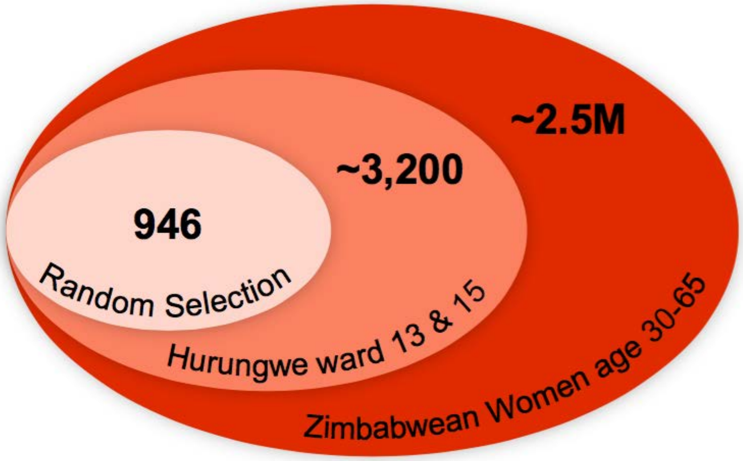

Figure 1. Population de l'étude en pourcentage de la population du district et du pays.

Les femmes ont été recrutées pour participer à des journées de sensibilisation communautaire, conduites en parallèle avec des campagnes programmées d'immunisations du PEV et de sensibilisation aux TAR dans 12 centres de villages. Les femmes recrutées au cours des 2-3 premiers mois de l'étude ont eu de nombreuses occasions de participer, alors que les occasions étaient moins nombreuses pour celles recrutées lors des cycles de mars et avril. Toutes les femmes ont reçu une savonnette (valeur <$1USD) en prime, même si elles n'ont pas été retenues (en raison de leur âge ou d'une grossesse en cours). Les femmes enceintes et les femmes âgées de moins de 30 ans ont été exclues, car on sait qu'elles présentent une prévalence accrue d'infections aux HPV-Hr avec des cycles d'élimination complexes qu'on n'observe pas chez les femmes qui ne sont pas enceintes [19, 20]. Les femmes âgées de moins de 30 ans ont des taux de prévalence d'infection aux HPV-Hr supérieurs, mais des taux inférieurs de détection de néoplasie intra-épithéliale cervicale (CIN) et de cancer du col de l'utérus[21]. Le statut sérologique du VIH n'a pas été utilisé comme critère d'inclusion ou d'exclusion.

Bien que le contexte de l'emploi formel soit difficile au Zimbabwe, les zones rurales sont un peu moins touchées grâce à l'agriculture vivrière, à la croissance annuelle des récoltes et à la vente saisonnière des cultures de rapport (tabac, coton, maïs). Les difficultés liées au pouvoir

d'achat ont résulté dans l'instauration d'un système de troc au sein des zones rurales (y compris pour les frais hospitaliers). La saison agricole particulièrement bonne durant la période de l'étude aurait pu faire baisser la participation en raison d'un besoin accru de main-d'œuvre.

Les prélèvements HPV-Hr ont été effectués dans la communauté durant les visites de sensibilisation pour la fourniture de TAR et la vaccination des enfants. Des agents de saisie des données formés ont donné des instructions pour l'auto-prélèvement sous forme orale, écrite et illustrée/infographique, et des sessions d'informations se sont tenues en Shona, la langue de l'ethnie la plus présente dans cette région du Zimbabwe. Les prélèvements ont ensuite été transportés avec les équipements médicaux et le personnel au Chidamoyo Christian Hospital pour subir un test utilisant le dispositif in vitro préqualifié par l'OMS, le Cepheid GeneXpert for HPV. Les spécimens cervicovaginaux auto-prélevés à l'aide d'une cytobrosse ont été obtenus avec des brosses imprégnées de solution ThinPrep PreservCyt (Hologic, Marlborough, MA). Le test Xpert® HPV a été conduit sur un GeneXpert (Cepheid, Sunnyvale, CA) basé en clinique, dans le respect des instructions du fabricant, dans les deux jours à une semaine suivant le prélèvement, en fonction de la disponibilité de la plate-forme de test et de l'électricité. Les spécimens présentant des résultats non valides ont été réexaminés. En cas de double échec d'un prélèvement, la participante était contactée pour effectuer un nouveau prélèvement lors d'une visite dans son village ou à l'occasion de son passage au Chidamoyo Christian Hospital.

En cas de dépistage positif au HPV-Hr, le coordinateur clinique du Chidamoyo prévenait les agents de santé communautaires et les femmes étaient invitées à une IVA à des dates définies. Il faut noter qu'il n'existait pas de programme d'IVA au Chidamoyo avant l'étude, mais trois infirmières et un docteur avaient suivi au moment de la conception de l'étude la formation à l'IVA dispensée par le Ministère de la santé, afin de fournir ces services à tous les membres de la communauté.

Analyse statistique

L'analyse statistique a été réalisée avec le logiciel STATA, v. 14 (College Station, TX). Une taille d'échantillon de 700 femmes a été calculée comme étant suffisante pour déterminer une différence pour l'infection/le type de HPV-Hr avec une puissance de 0,80, une taille d'effet de 0,25 et un niveau alpha de 0,05 avec un échantillonnage proportionné des villages de tailles

différentes permettant de fournir un échantillon représentatif de la population. L'analyse de participation basique a été réalisée avec les données saisies dans un tableur Excel et dans le logiciel STATA, v. 14, avec les catégories “non-présentation”, “ participation”, “inéligible” ou “refus”. Les données démographiques concernaient uniquement les femmes qui ont participé à l'étude via ODK sur des tablettes, et les résultats ont été téléchargés sous forme de tableurs Excel et les données démographiques de base ont été analysées dans STATA, v. 14.

Configuration de l'étude

Le Zimbabwe, situé en Afrique australe, est frontalier du Botswana, de la Zambie, du Mozambique et de l'Afrique du Sud. La population du Zimbabwe est estimée à 14 millions d'habitants, vivant principalement dans des zones rurales [22]. La prévalence du VIH au Zimbabwe est estimée à environ 14,6 % [6], et légèrement plus dans la population adulte, il affecte les femmes de manière disproportionnée, avec un taux de trois femmes pour deux hommes[23]. À une époque, le taux d'alphabétisation du pays était le plus élevé d'Afrique subsaharienne – en 2015, 86,5 % de la population avaient des connaissances de base[22]. Le taux d'emploi après la crise d'hyperinflation de 2007 reste faible, avec un taux d'inactivité estimé entre 50 et 90 %, toutefois, des estimations précises sont impossibles en l'état actuel de la conjoncture [22].

Chidamoyo Christian Hospital

L'étude a été conduite dans les zones rurales du nord-ouest du Zimbabwe, dans le district de Hurungwe, situé dans la province du Mashonaland occidental. La zone de l'étude a été définie comme les municipalités (Ward) 13/15, dans la zone desservie par le Chidamoyo Christian Hospital, qui englobe une population totale estimée à 32 000 personnes.

Le Chidamoyo Christian Hospital dispose de 100 lits, d'un bloc chirurgical, d'une salle de travail et d'accouchement, d'une unité pédiatrique et d'une clinique de jour gérée par une infirmière praticienne (auteur KM), 1 à 3 médecins zimbabwéens et une vingtaine d'infirmiers, dont la plupart sont des “infirmiers auxiliaires” formés sur place. L'hôpital coordonne les campagnes d'immunisation au sein du PEV initié en 1974 par l'OMS pour la vaccination des

enfants à l'échelle mondiale. Le modèle médical communautaire a revêtu une forme totalement nouvelle à l'ère du VIH dans le programme de sensibilisation du Chidamoyo au Hurungwe. En plus d'administrer les vaccins du PEV aux enfants, le Chidamoyo dispense également des traitements antirétroviraux (TAR) aux patients infectés par le VIH lors de visites communautaires hebdomadaires programmées sur chaque site tous les deux mois.

Formation des agents de santé communautaires

Au cours de trois ateliers d'une journée, les agents de santé communautaires ont reçu une formation sur la prévention du cancer du col de l'utérus et sur le test de dépistage ADN HPV, les méthodes et objectifs de l'étude. Des guides illustrés, une formation sur PowerPoint et des interactions pratiques au sein de petits groupes ont aidé les ASC à comprendre le cancer du col de l'utérus, le dépistage des HPV et la conception de l'étude. Les agents de santé communautaires ont fourni des listes de toutes les femmes éligibles de leurs villages deux mois avant le début de l'étude. Trois membres de la communauté ont été formés à la saisie des données et à la dispense des instructions pour les méthodes d'auto-prélèvement. Les agents de santé communautaires femmes ont reçu un auto-test HPV pour gagner en expérience et comme service communautaire, bien qu'elles n'aient pas été incluses dans les données de l'étude. Les agents de santé communautaires ont informé les chefs de villages, les directeurs d'écoles et d'autres responsables de communautés, ainsi que le Ministère de la santé (MS), avant de lancer l'étude. Les agents de santé communautaires ont été informés des femmes sélectionnées au hasard dans leur village, et les ont prévenues lors de visites au domicile pour leur expliquer les objectifs de l'étude et encourager la participation aux journées de collecte communautaire au sein de leurs villages respectifs.

Dépistage du VIH.

Un dépistage sérologique du VIH a été effectué par des conseillers VIH certifiés du ministère de la santé, au moyen du test de 3e génération Alere Determine HIV-1/2 (Alere/Abbott, Lake Bluff, Illinois, U.S.) fourni par le Ministère de la santé, un test immunologique de qualité permettant la détection d'anticorps au VIH-1 et VIH-2. Les spécimens positifs ont été confirmés avec le test rapide sur bandelette du VIH 1-2 First Response (Premier

Medical Corporation Ltd., Kachigam, Inde), qui est un test immuno-chromatographique qualitatif pour la détection des anticorps aux VIH-1 et VIH-2 dans le sang entier, le sérum ou le plasma. Les résultats étaient interprétés, conformément aux instructions du fabricant, par des conseillers VIH formés.

Résultats

Globalement, le taux de réponse à l'étude a été de 82 % (N=778/946), avec 12 % de femmes inéligibles car âgées de moins de 30 ans ou de plus de 65 ans, ou enceintes (N=117/946). Parmi les 4 % (N=40/946) qui ont refusé de participer, le motif le plus fréquemment invoqué était les croyances religieuses (42 % ; N=17/40). Un déménagement, la peur des résultats et/ou de l'examen pelvien, et la pression ou l'interdiction par le mari comptent parmi les autres motifs. Seulement 8 % des femmes incluses dans le recrutement initial (groupe recruté en premier) ne se sont pas présentées ou ont refusé le dépistage (5 % de refus, 3 % d'absences). Très peu d'échantillons étaient techniquement non valides en raison d'un prélèvement inadéquat ou d'une difficulté technique (6/654, 0,9 %). Les premières femmes recrutées pour l'étude avec le plus d'occasions de participer (moyenne de 3 visites de la communauté) ont présenté un taux de participation significativement supérieur : 81 % et 11 % d'inéligibles, tandis que celles avec moins d'occasions ont également moins participé : 63 % et 13 % d'inéligibles ($p<000,1$). La participation a également été inférieure durant certaines parties de la saison des pluies (de novembre à avril) et au début de la saison des récoltes (mai à juin).

Certains centres communautaires (N=5/12) ont présenté une participation supérieure à 89 % des femmes présentes sur les listes, avec de nombreuses femmes non incluses dans l'étude demandant chaque jour de participer. Les villages à distance de marche du Chidamoyo Hospital avec des agents de santé communautaires très actifs ont obtenu des taux de participation allant de 89 à 93 %. Dans d'autres zones, plus éloignées de l'hôpital et avec des agents de santé communautaires moins actifs, les taux de participation n'ont été que de 75 à 81 % (Figure 2).

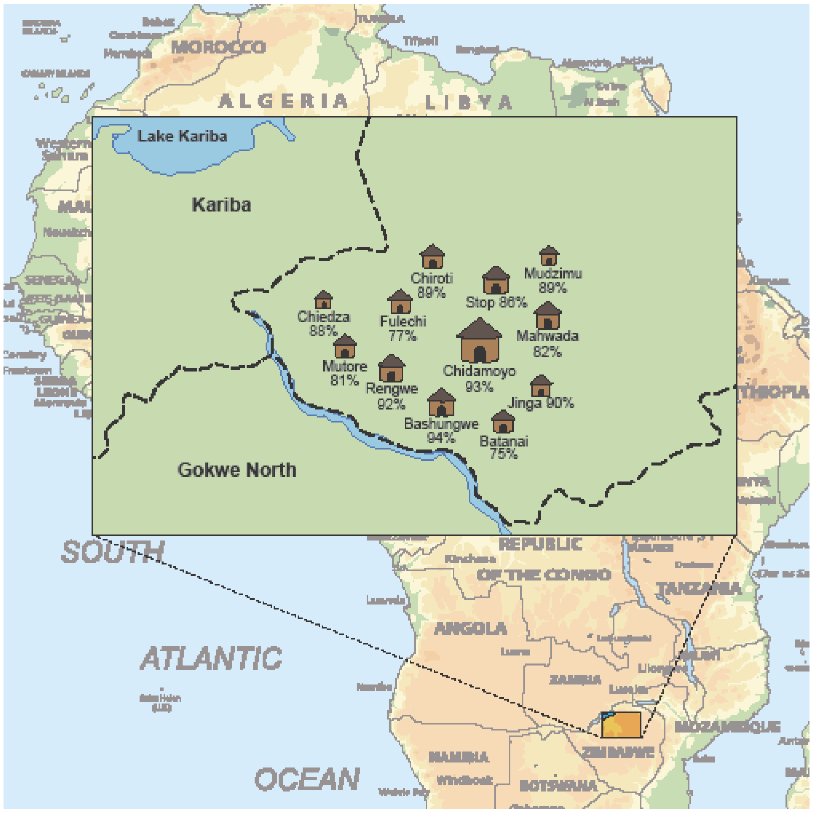

Figure 2. Plan des postes de contact des villages dans les municipalités 13 et 15 du district de Hurungwe (Zimbabwe), avec les taux de participation à l'étude. Chidamoyo est le centre hospitalier.

L'âge moyen des participants à l'étude était de 43,6 ans, l'âge moyen à la première grossesse était de 19,0 ans et le nombre de grossesses de 4,47. La moitié (49,5 %) des femmes avaient un niveau d'éducation équivalent à la classe de 5e, tandis que 24 % n'avaient aucune éducation scolaire formelle. Aucune n'avait suivi d'éducation supérieure. La grande majorité des femmes (95,1 %) n'avaient jamais été dépistées pour le cancer du col de l'utérus. La prévalence du VIH était de 21,8 % dans la population totale testée (0,3 % de statut inconnu). La prévalence du VIH était la plus basse parmi les femmes âgées de plus de 50 ans (15 %), tandis que les femmes de moins de 40 ans présentaient les taux les plus élevés de détection des HPV-HR (20 %) (figure 3). De nombreuses femmes séropositives pour le VIH prenaient déjà des TAR au moment de l'étude (90 %).

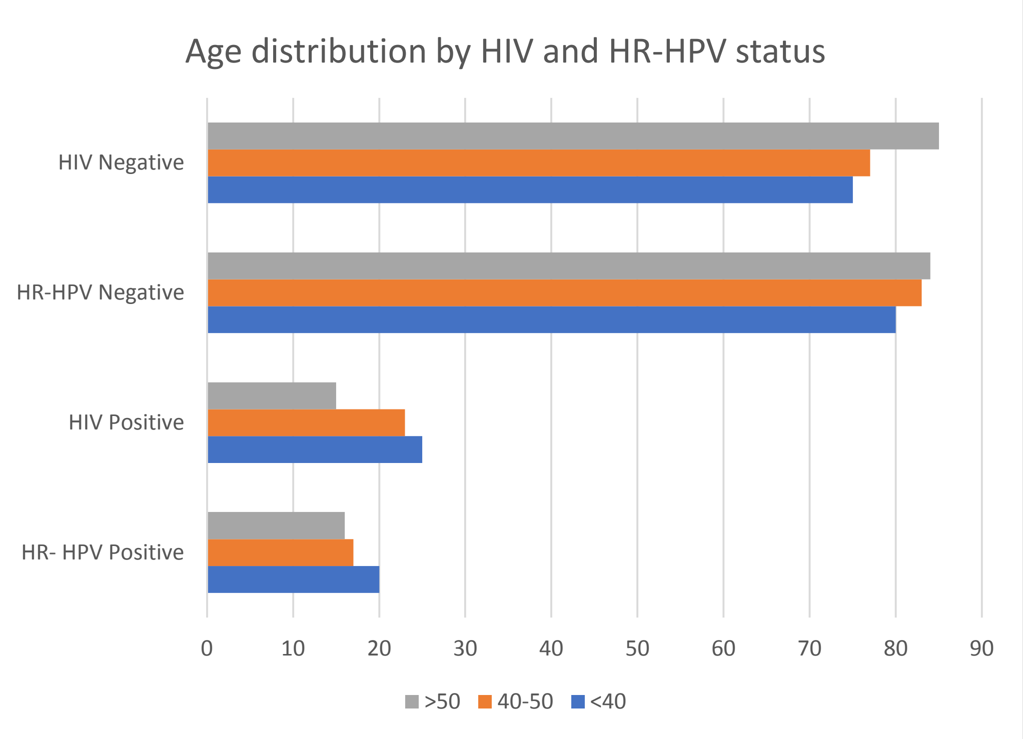

Figure 3. Ce graphique indique la répartition par catégorie d'âge.

Discussion

L'Afrique subsaharienne est la région la plus touchée par le VIH ; l'Afrique orientale et l'Afrique australe représentent 43 % des nouvelles infections au niveau mondial et on estime à 19,4 millions le nombre de séropositifs dans la région, dont 21,8 % de femmes dans notre étude [23]. Les données provenant de l'enquête démographique et sanitaire de 2015 au Zimbabwe ont montré que 14 % des adultes (15-49 ans) étaient porteurs du VIH au Zimbabwe, avec une prévalence plus élevée parmi les femmes (17 %) que parmi les hommes (11 %)[24]**.** Les femmes séropositives ont un risque plus élevé d'infection aux HPV-Hr[25] et de développer un cancer du col de l'utérus[26]. Alors que le dépistage du cancer du col de l'utérus est essentiel chez les femmes infectées au VIH, il n'est actuellement pas intégré aux soins liés au VIH.

Les programmes communautaires de dépistage et de traitement pour le VIH ont accru l'amorce de traitements à base d'antirétroviraux et de suppression de la charge virale dans des lieux aux ressources limitées [27]. L'hôpital Chidamoyo a commencé à combiner ses services de sensibilisation aux TAR avec les campagnes de vaccinations des enfants en 2006. Depuis lors,

d'autres services de santé sexuelle et reproductive, tels que le planning familial, ont été intégrés avec succès dans ces modèles de sensibilisation existants. L'intégration du dépistage des HPV-Hr au sein de ce cadre pourrait accroître l'accès et le recours aux services de dépistage du cancer du col de l'utérus. Dans notre étude descriptive, 95 % des femmes rurales n'avaient jamais été dépistées pour le cancer du col de l'utérus au moyen d'approches cliniques (tableau 1), tandis que plus de 82 % des femmes ont participé au dépistage communautaire.

Des vaccinations efficaces contre le papillomavirus humain sont disponibles et seront bientôt accessibles aux Zimbabwe grâce à un partenariat avec l'Alliance mondiale pour les vaccins et la vaccination (GAVI) initié en mai 2018[28]. Enfin, combiner le dépistage du (pré)cancer et la prévention primaire via la vaccination est crucial, car ces vaccins disponibles sont conçus pour éviter l'infection par les HPV chez les jeunes, et laissent une proportion considérable de la population adulte vulnérable. De plus, des sous-types de HPV autres que les HPV-Hr 16/18 sont connus pour circuler et causer le cancer en Afrique, et peuvent continuer à représenter un risque malgré la vaccination [3, 5, 25, 29, 30]. Par ailleurs, les femmes adultes porteuses du VIH présentent un risque supérieur de cancer du col de l'utérus et requièrent une surveillance continue [25, 26, 30, 31].

Notre programme illustre une méthode potentielle pour atteindre les femmes en vue du dépistage. Le dépistage des HPV-Hr sur les lieux de soins pourrait être combiné à des programmes communautaires existants, afin d'améliorer la prévention du cancer en Afrique subsaharienne chez les femmes les plus à risque [16].

Limitations de l'étude

Les taux de participation au dépistage par auto-prélèvement via la sensibilisation dans les villages étaient globalement prometteurs vus les taux de dépistage de référence (5 % vs. 60-80 %). Notre étude ne disposait pas d'une population témoin permettant de comparer les taux de participation, et elle a été conduite dans une région qui ne proposait pas le dépistage du cancer de l'utérus avant l'étude. En outre, ne disposant pas d'un groupe de comparaison, nous n'avons pas pu exclure complètement les facteurs parasites qui ont pu accroître ou réduire la participation au dépistage.

Conclusions

Nos résultats suggèrent que l'intégration du dépistage communautaire des HPV-Hr par auto-prélèvement dans les programmes de traitement du VIH et de vaccinations des enfants pourrait être considérée comme une méthode potentielle permettant d'étendre les programmes de dépistage du cancer du col de l'utérus dans les zones rurales difficiles à atteindre.

Liste des abréviations

PRFI : pays à revenus faibles et intermédiaires
HPV-Hr : papillomavirus à haut risque
VIH : virus de l'immunodéficience humaine
TAR : traitement antirétroviral
OMS : Organisation mondiale de la Santé
USD : dollar US
IVA : inspection visuelle avec l'acide acétique et cervicographie
PEV : Programme élargi de vaccination
ASC : agent de santé communautaire *terme interchangeable avec agent de santé rural
ASR : agent de santé rural

Concernant ce supplément

Cet article a été publié comme partie de l'ouvrage de *BMC Public Health*, Volume 19 Supplement 1, 2019: Effective Integration of Sexual Reproductive Health and HIV Prevention, Treatment, and Care Services across sub-Saharan Africa: Where is the evidence for program implementation?
L'intégralité du contenu, avec les versions en français, en portugais et en anglais, est disponible en ligne :
https://bmcpublichealth.biomedcentral.com/articles/supplements/volume-19-supplement-1
et
https://reproductive-health-journal.biomedcentral.com/articles/supplements/volume-16-supplement-1

Déclarations

Approbation éthique et accord de participation

L'approbation éthique a été accordée par l'Université de Stanford (n° 37975), l'Université du Zimbabwe (JREC 221/16), le Conseil pour la recherche médicale du Zimbabwe (MRCZ/A/2128) et le Conseil pour la recherche du Zimbabwe (n° 02921). En outre, les médecins-conseils de la Province et du District ont été notifiés, ainsi que les chefs de villages durant les réunions communautaires, après la sensibilisation via la formation des agents de santé communautaires préalablement à la saisie des données. Les femmes ont été informées que leur participation

était libre et qu'elles pouvaient se retirer à tout moment, que nous leur proposerions un test de dépistage du VIH mais qu'elles pouvaient refuser ce test ou refuser d'en connaître le résultat, et que toutes les informations concernant leur statut sérologique pour le VIH et les HPV resteraient confidentielles. La plupart des femmes ont voulu connaître les résultats des tests de dépistage du VIH et des HPV. Les femmes éligibles ont été interrogées oralement par des agents de saisie des données de recherche formés, membres de l'équipe de recherche, au moyen d'un questionnaire électronique permettant de collecter des informations de nature sociodémographique et reproductive. L'inclusion a eu lieu après signature d'un consentement éclairé individuel par voie électronique ou par empreinte digitale sur un exemplaire papier pour les personnes illettrées, et un autre exemplaire papier était donné à la participante. Le consentement éclairé (signature ou empreinte digitale en présence d'un témoin) a été obtenu auprès de toutes les participantes avant leur inclusion.

Accord de publication
Toutes les informations à caractère personnel ont été anonymisées, et l'obtention d'un accord de publication n'est donc pas applicable.

Disponibilité des données et matériels
Toutes les données anonymisées sont disponibles pour le public sur demande.

Conflits d'intérêts
Les auteurs déclarent ne pas avoir de conflits d'intérêts.

Financement
Cette étude a été financée par une bourse de formation des National Institutes of Health Fogarty pour l'équité mondiale en termes de santé, accordée à MBF, sous la référence TW0009338 R25, et par une Bourse à la formation encadrée du Service de pathologie de Stanford, octroyée à MBF et à PAB. La publication de cet article a été financée par le Service de pathologie de Stanford.

Le supplément de la revue est rendu possible grâce au soutien généreux du peuple américain via la United States Agency for International Development (USAID) en partenariat avec le Fonds des Nations unies pour la population (FNUAP) et le Programme commun des Nations Unies sur le VIH/SIDA (ONUSIDA).

Les opinions exprimées dans la présente publication sont celles des auteurs et ne reflètent pas nécessairement les politiques officielles de l'USAID, du FNUAP ou de l'ONUSIDA, la mention des dénominations de ministères ou d'organismes n'implique pas non plus l'aval du gouvernement américain, du FNUAP ou de l'ONUSIDA.

Contributions des auteurs
Rôles et responsabilités des auteurs : MBF a été impliqué dans la conception de l'étude, les approbations de l'IRB, la demande de subvention, la saisie des données, l'analyse des données, la première ébauche du manuscrit, les principales modifications du manuscrit ; ZEK a été impliqué dans la conceptualisation du manuscrit et les principales modifications du manuscrit ;

DK a été impliqué dans la conceptualisation de l'étude et du manuscrit, la demande de subvention, l'analyse des données, les principales modifications du manuscrit ; BAP a été impliqué dans la conceptualisation de l'étude et du manuscrit, la demande de subvention, la saisie des données, l'analyse des données, les principales modifications du manuscrit ; KM a été impliqué dans la conceptualisation de l'étude, la saisie des données, l'analyse des données et la modification du manuscrit. Tous les auteurs ont lu et approuvé le manuscrit final.

Remerciements
Nous remercions le Chidamoyo Christian Hospital pour sa coopération et son aide dans tous les aspects de cette étude. En particulier, le coordinateur de l'étude, Edwell Mereki, les agents de saisie des données : Semya Mereki, Christine Momemebere, Nancy Momembere, le technicien de laboratoire : Oliver Sakawaya, l'administrateur de l'hôpital : Major Mereki, ainsi que le personnel infirmier et les médecins. Nous remercions également le Professeur Zavahera Mike Chirenje pour son aide dans la conceptualisation du projet et pour sa contribution par ses approbations et par la mise en œuvre du projet. Nous remercions l'Université du Zimbabwe pour son aide au niveau du laboratoire, en particulier Fiona Mutisi. Nous remercions Laurel Stell pour le traitement statistique à Stanford. De plus, nous remercions Bhavini Suraiya Varyani, Justen Manasa et Vinie Kouamou pour leur aide logistique et leur assistance au laboratoire. Enfin, nous remercions Raymond Chibvongodze et Rama Arumilli pour l'examen des frottis ThinPrep Pap. Une aide matérielle a été fournie par Cepheid qui a fait don de 600 cartouches HPV, et par Hologic, Inc. qui a fait don de 600 tubes de prélèvement ThinPrep et de 500 filtres pour cytologie ThinPrep.

Références

1. Chidyaonga-Maseko F, Chirwa ML, Muula AS. Underutilization of cervical cancer prevention services in low and middle income countries: a review of contributing factors. Pan Afr Med J. 2015;21. doi:10.11604/pamj.2015.21.231.6350.

2. Verdoodt F, Jentschke M, Hillemanns P, Racey CS, Snijders PJF, Arbyn M. Reaching women who do not participate in the regular cervical cancer screening programme by offering self-sampling kits: A systematic review and meta-analysis of randomised trials. Eur J Cancer. 2015;51:2375–85.

3. Baay MFD, Kjetland EF, Ndhlovu PD, Deschoolmeester V, Mduluza T, Gomo E, et al. Human papillomavirus in a rural community in Zimbabwe: The impact of HIV co-infection on HPV genotype distribution. J Med Virol. 2004;73:481–5.

4. World Health Organization (WHO). Prevention of cervical cancer through screening using visual inspection with acetic acid (VIA) and treatment with cryotherapy A demonstration project in six African countries: (Malawi, Madagascar, Nigeria, Uganda, the United Republic of Tanzania, and Zambia). 2012. http://apps.who.int/iris/bitstream/10665/75250/1/9789241503860_eng.pdf?ua=1. Accessed 13 Dec 2016.

5. Chin'ombe N, Sebata NL, Ruhanya V, Matarira HT. Human papillomavirus genotypes in cervical cancer and vaccination challenges in Zimbabwe. Infect Agent Cancer. 2014;9:16.

6. ZIMBABWE POPULATION-BASED HIV IMPACT ASSESSMENT. http://phia.icap.columbia.edu/wp-content/uploads/2016/11/ZIMBABWE-Factsheet.FIN_.pdf. Accessed 19 Dec 2017.

7. Franke MF, Kaigamba F, Socci AR, Hakizamungu M, Patel A, Bagiruwigize E, et al. Improved retention associated with community-based accompaniment for antiretroviral therapy delivery in rural Rwanda. Clin Infect Dis Off Publ Infect Dis Soc Am. 2013;56:1319–26.

8. Wouters E, Van Damme W, van Rensburg D, Masquillier C, Meulemans H. Impact of community-based support services on antiretroviral treatment programme delivery and outcomes in resource-limited countries: a synthetic review. BMC Health Serv Res. 2012;12:194.

9. Busza J, Dauya E, Bandason T, Simms V, Chikwari CD, Makamba M, et al. The role of community health workers in improving HIV treatment outcomes in children: lessons learned from the ZENITH trial in Zimbabwe. Health Policy Plan. 2018;33:328–34.

10. Torpey KE, Kabaso ME, Mutale LN, Kamanga MK, Mwango AJ, Simpungwe J, et al. Adherence Support Workers: A Way to Address Human Resource Constraints in Antiretroviral Treatment Programs in the Public Health Setting in Zambia. PLoS ONE. 2008;3. doi:10.1371/journal.pone.0002204.

11. Walboomers JMM, Jacobs MV, Manos MM, Bosch FX, Kummer JA, Shah KV, et al. Human papillomavirus is a necessary cause of invasive cervical cancer worldwide. J Pathol. 1999;189:12–9.

12. World Health Organization, editor. WHO guidelines for screening and treatment of precancerous lesions for cervical cancer prevention. Geneva: World Health Organization; 2013.

13. Bansil P, Wittet S, Lim JL, Winkler JL, Paul P, Jeronimo J. Acceptability of self-collection sampling for HPV-DNA testing in low-resource settings: a mixed methods approach. BMC Public Health. 2014;14:1.

14. Arbyn M, Verdoodt F, Snijders PJF, Verhoef VMJ, Suonio E, Dillner L, et al. Accuracy of human papillomavirus testing on self-collected versus clinician-collected samples: a meta-analysis. Lancet Oncol. 2014;15:172–83.

15. Petignat P, Faltin DL, Bruchim I, Tramèr MR, Franco EL, Coutlée F. Are self-collected samples comparable to physician-collected cervical specimens for human papillomavirus DNA testing? A systematic review and meta-analysis. Gynecol Oncol. 2007;105:530–5.

16. Ibáñez R, Autonell J, Sardà M, Crespo N, Pique P, Pascual A, et al. Protecting the underscreened women in developed countries: the value of HPV test. BMC Cancer. 2014;14. doi:10.1186/1471-2407-14-574.

17. Untiet S, Vassilakos P, McCarey C, Tebeu P-M, Kengne-Fosso G, Menoud P-A, et al. HPV self-sampling as primary screening test in sub-Saharan Africa: implication for a triaging strategy. Int J Cancer. 2014;135:1911–7.

18. Arrossi S, Thouyaret L, Herrero R, Campanera A, Magdaleno A, Cuberli M, et al. Effect of self-collection of HPV DNA offered by community health workers at home visits on uptake of screening for cervical cancer (the EMA study): a population-based cluster-randomised trial. Lancet Glob Health. 2015;3:e85–94.

19. Nobbenhuis MAE, Helmerhorst TJM, van den Brule AJC, Rozendaal L, Bezemer PD, Voorhorst FJ, et al. High-risk human papillomavirus clearance in pregnant women: trends for lower clearance during pregnancy with a catch-up postpartum. Br J Cancer. 2002;87:75–80.

20. Salcedo MMBP, Damin APS, Agnes G, Pessini SA, Beitune PE, Alexandre COP, et al. Prevalence of human papillomavirus infection in pregnant versus non-pregnant women in Brazil. Arch Gynecol Obstet. 2015;292:1273–8.

21. Liu Z, Li Q, Li W. Epidemiological studies of women under age 30 infected with human papillomavirus. Front Med China. 2009;3:459–62.

22. The World Factbook — Central Intelligence Agency. https://www.cia.gov/library/publications/the-world-factbook/geos/zi.html. Accessed 30 Nov 2017.

23. UNAIDS_FactSheet_en.pdf. http://www.unaids.org/sites/default/files/media_asset/UNAIDS_FactSheet_en.pdf. Accessed 29 Nov 2017.

24. Health and Vital Surveys. http://www.nada.zimstat.co.zw/nada/index.php/catalog/HLT/about. Accessed 5 Aug 2018.

25. Adler D, Wallace M, Bennie T, Abar B, Sadeghi R, Meiring T, et al. High Risk Human Papillomavirus Persistence Among HIV-infected Young Women in South Africa. Int J Infect Dis IJID Off Publ Int Soc Infect Dis. 2015;33:219–21.

26. Chaturvedi AK, Madeleine MM, Biggar RJ, Engels EA. Risk of Human Papillomavirus–Associated Cancers Among Persons With AIDS. JNCI J Natl Cancer Inst. 2009;101:1120–30.

27. Petersen M, Balzer L, Kwarsiima D, Sang N, Chamie G, Ayieko J, et al. Association of Implementation of a Universal Testing and Treatment Intervention With HIV Diagnosis, Receipt of Antiretroviral Therapy, and Viral Suppression in East Africa. JAMA. 2017;317:2196–206.

28. Cervical cancer vaccine introduced in Zimbabwe. https://www.gavi.org/library/news/press-releases/2018/cervical-cancer-vaccine-introduced-in-zimbabwe/. Accessed 29 Oct 2018.

29. Howitt BE. Comprehensive Human Papillomavirus Genotyping in Cervical Squamous Cell Carcinomas and Its Relevance to Cervical Cancer Prevention in Malawian Women. J Glob Oncol. 2017;3. http://sfx.stanford.edu/local?sid=stanford:laneweb-search-pubmed&id=pmid:28717764. Accessed 8 Sep 2017.

30. Maranga IO, Hampson L, Oliver AW, He X, Gichangi P, Rana F, et al. HIV infection alters the spectrum of HPV subtypes found in cervical smears and carcinomas from Kenyan Women. Open Virol J. 2013;7. http://benthamopen.com/FULLTEXT/TOVJ-7-19. Accessed 26 Sep 2016.

31. Kelly H, Weiss HA, Benavente Y, Sanjose S de, Mayaud P, Qiao Y, et al. Association of antiretroviral therapy with high-risk human papillomavirus, cervical intraepithelial neoplasia, and invasive cervical cancer in women living with HIV: a systematic review and meta-analysis. Lancet HIV. 2018;5:e45–58.

Tableau 1.

Caractéristiques démographiques des participantes à l'étude

**Tableau 1. Caractéristiques des participantes à l'étude (N=683)**	
**Âge (années ± écart-type)**	43,6 (10,2)
**Âge à la première grossesse (années ± écart-type)**	19,0 (3,2)
**Nombre de grossesses (moyenne ± écart-type)**	4,47 (2,4)
**Éducation**	
**Aucune scolarisation**	163 (24,0 %)
**4 années**	177 (26,1 %)
**6 années**	3 (0,4 %)
**7 années**	336 (49,5 %)
**Études supérieures**	0 (0,0 %)
**Historique de la contraception hormonale**	480 (70,8 %)
**Dépistage préalable du cancer du col de l'utérus**	
**Non (pas de dépistage préalable)**	646 (95,1 %)
**Oui (dépistage préalable)**	33 (4,9 %)
**Catégories d'âges**	
**30-39**	302 (44,5 %)
**40-49**	186 (27,4 %)
**50-65**	191 (28,1 %)
**Statut VIH**	
**Séropositif**	146 (21,8 %)
**Séronégatif**	521 (77,9 %)
**Inconnu**	2 (0,3 %)
