# Supplementary material for: Community-based self-collected human papillomavirus screening in rural Zimbabwe
Source: BMC Public Health. 2019 May 29;19(Suppl 1):603. doi: 10.1186/s12889-019-6810-5 (PMC6538544; doi:10.1186/s12889-019-6810-5)
Supplement: Supplementary file 2 — Translation of the abstract of this article into Portuguese. (PDF 99 kb) [file 12889_2019_6810_MOESM2_ESM.pdf]

BMC Public Health 2019, 19 (Suppl 1) [translated from the original publication]

Rastreio comunitário do vírus do papiloma humano, recolhido pelas próprias pessoas, na zona rural do Zimbabué

Megan B. Fitzpatrick[1*], Ziad El-Khatib [2,3], David Katzenstein[4,5], Benjamin A. Pinsky[1,4], Zvavahera Mike Chirenje[6], Kathy McCarty[7]

[1]Stanford University School of Medicine, Department of Pathology, 300 Pasteur Drive, Stanford, CA, 94305, USA; megan6@stanford.edu, davidkk@stanford.edu, bpinsky@stanford.edu
[2]Department of Public Health Sciences, Karolinska Instiutet, Stockholm, Sweden
[3]World Health Programme, Université du Québec en Abitibi-Témiscamingue (UQAT), Québec, Canada, ziad.khatib@gmail.com
[4]Stanford University School of Medicine, Department of Medicine, Division of Infectious Diseases and Geographic Medicine, 300 Pasteur Drive, Stanford, CA 94305, USA
[5]Biomedical Research and Training Institute of Zimbabwe, 10 Seagrave Rd, Mount Pleasant, Harare Zimbabwe
[6]University of Zimbabwe, Department of Obstetrics and Gynecology, 630 Churchill Avenue, Harare, Zimbabwe; mchirenje@uzchs-ctu.org
[7]Chidamoyo Christian Hospital, P.O. Box 330, Karoi, Zimbabwe; sistermakate@gmail.com

* Autor correspondente: Dr. Megan Fitzpatrick
E-mail: megan6@stanford.edu

Resumo

Introdução: Nos países de baixo rendimento (PRMI), as mulheres têm acesso limitado ao rastreio do cancro do colo do útero. O diagnóstico tardio conduz a piores resultados e à mortalidade precoce e continua a impedir o controlo do cancro de forma desproporcional nos PRMI. A integração do rastreio comunitário do vírus do papiloma humano (HPV) de alto risco, recolhido pelas próprias pessoas, em programas existentes do VIH é um possível método de rastreio para identificar mulheres em alto risco de desenvolverem lesões do colo do útero de alto risco.

Métodos: Foi implementado um estudo transversal na comunidade sobre o rastreio do HPV de alto risco, recolhido pelas próprias pessoas, em articulação com os programas comunitários de proximidade existentes para distribuição do tratamento antirretroviral (TAR) e o Programa Expandido de Imunizações (PEI) da Organização Mundial de Saúde nas aldeias da zona rural do Zimbabué, de janeiro de 2017 a maio de 2017.

Resultados: No geral, houve uma taxa de resposta de 82%: 70% dos inquiridos participaram na recolha efetuada pelas próprias e 12% foram inelegíveis para o estudo (critérios de inclusão: mulheres com idades compreendidas entre 30 e 65 anos, não grávidas e com o útero intacto). As mulheres recrutadas entre os primeiros dois e três meses do estudo tiveram mais oportunidades para participar, o que significa que tiveram um nível de participação significativamente superior: 81% de participação (mais 11% inelegíveis), enquanto que as mulheres com menos oportunidades também tiveram um nível de participação inferior: 63% (mais 13% inelegíveis) ($p<0,001$). Alguns centros de proximidade das aldeias (n=5/12) tiveram um nível de participação superior a 89%.

Conclusões: A integração do rastreio do HPV de alto risco em programas comunitários de proximidade existentes para o VIH e imunizações pode facilitar o rastreio populacional de forma a aumentar os programas de prevenção e controlo do cancro na África Subsariana. Os profissionais de saúde das comunidades/aldeias (PSC/PSA) e os programas de proximidade das aldeias oferecem uma possível opção para os programas de rastreio do cancro do colo do útero melhorarem o acesso a recursos de saúde sexual e reprodutiva para as mulheres em alto risco.

Palavras-chave
Vírus do papiloma humano, Rastreio do cancro do colo do útero, Rastreio do cancro

Sobre este suplemento

Este resumo foi publicado como parte da revista científica *BMC Public Health*, Volume 19, Suplemento 1, 2019: Integração Eficaz dos Serviços de Saúde Sexual e Reprodutiva e de Prevenção, Cuidados e Tratamento do VIH na África Subsariana: Onde estão as provas da implementação do programa?
O suplemento foi publicado como uma colaboração entre as revistas científicas *Reproductive Health* e *BMC Public Health.* O conteúdo integral, incluindo as versões em francês, português e inglês, estão disponíveis online:
https://bmcpublichealth.biomedcentral.com/articles/supplements/volume-19-supplement-1
e
https://reproductive-health-journal.biomedcentral.com/articles/supplements/volume-16-supplement-1
